# A Comprehensive Survey on Resource Allocation Strategies in Fog/Cloud Environments

**DOI:** 10.3390/s23094413

**Published:** 2023-04-30

**Authors:** Jaime Vergara, Juan Botero, Luis Fletscher

**Affiliations:** Faculty of Engineering, Universidad de Antioquia, Medellín 50010, Colombia; juanf.botero@udea.edu.co (J.B.); luis.fletscher@udea.edu.co (L.F.)

**Keywords:** fog computing, cloud computing, resource allocation, IoT

## Abstract

The growing number of connected objects has allowed the development of new applications in different areas. In addition, the technologies that support these applications, such as cloud and fog computing, face challenges in providing the necessary resources to process information for different applications due to the highly dynamic nature of these networks and the many heterogeneous devices involved. This article reviews the existing literature on one of these challenges: resource allocation in the fog–cloud continuum, including approaches that consider different strategies and network characteristics. We also discuss the factors influencing resource allocation decisions, such as energy consumption, latency, monetary cost, or network usage. Finally, we identify the open research challenges and highlight potential future directions. This survey article aims to serve as a valuable reference for researchers and practitioners interested in the field of edge computing and resource allocation.

## 1. Introduction

According to the United Nations, in its review of global urbanization prospects, by 2018, 55% of the population will reside in urban areas, and by 2050, this percentage will grow to 68% [1]. In addition, Gartner [2] estimated that, by 2020, around twenty billion connected devices would be deployed, from end-user equipment, such as phones, tablets, and computers, to municipal systems for traffic management, climate, and health services [3]. There is also an increasing number of smart city initiatives being promoted to address urbanization challenges, including, for example, the European Commission’s Digital Agenda that focuses on the deployment of energy-efficient cities [4], the i-Japan strategy [5], Singapore’s Smart Nation 2015 plan [6], and the investment conducted by China and India in the implementation of more than 300 smart cities [7]. These are all clear examples of the interest of nations in not only the implementation but also in researching the deployment of intelligent territories, with a large number of connected objects (IoT) and technologies supporting applications that serve different actors. A key part of the operation of these solutions is the data life cycle, which starts and ends in the layer of interconnected objects. This requires the construction of a flexible architecture that allows data flow processing coming from different sources with different characteristics and particular QoS requirements.

In order to provide suitable solutions to the different requirements of the applications, new computing paradigms have been adopted in these architectures. Previously, sending data to applications located in the cloud using the Internet was a standard scheme. However, new IoT applications and the increasing data generated by users have shown the limitations of this alternative, especially when dealing with delay-sensitive applications [8]. Therefore, deploying computing resources closer to the sources could lead to achieving adequate service levels for every application. In this way, new paradigms such as mobile edge computing (MEC), mobile cloud computing (MCC), edge computing, cloudlets, and fog computing have shown that it is possible to reduce response time and bandwidth consumption, improve privacy and security conditions, facilitate the deployment of location-based services, and alleviate connectivity problems in environments that are hostile to connected objects, compared to systems deployed in the cloud [8].

**Figure 1:**
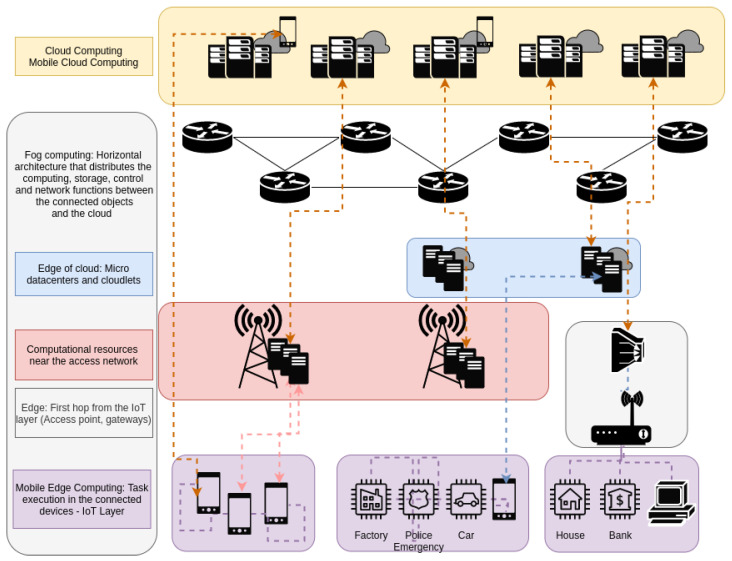
Cloud and fog integration.

The scope of each of these paradigms can be seen in Figure 1, where, for example, MEC tries to assign tasks to end devices, something that could be extended not only to mobile phones but also to vehicles or IoT devices. Another common option is exploiting resources on the first hop from the IoT layer in devices such as access points (APs) or access gateways, which generally provide more stable connections and higher computational resources. Some recent alternatives have implemented small servers near the cellular base stations to deploy content delivery networks (CDNs) in order to provide multimedia with lower latency to end users. Finally, there is a growing interest in deploying small data centers closer to the IoT layer, known as cloudlets; several works have included real implementations with commercial software where authors analyze the performance of solutions that perform resource allocation in these environments [9,10]. In this work, we will use the term fog for all the nodes that lay between the IoT layer and the cloud layer (including IoT nodes).

Due to the growing interest in developing solutions that include intermediate computation paradigms, this work devotes its attention to analyzing resource allocation problems in architectures that have computational resources distributed between the cloud and connected objects. Different articles [11,12,13,14] denominate these nodes as fog nodes (FNs) and are associated with a hierarchical architecture known as fog computing, which serves specific problems that cannot be solved using only traditional cloud-based solutions [15]. According to the Industrial Internet Consortium [16], these are nodes that have enough computational and network capacity to run advanced services, represented in edge devices such as connected vehicles, surveillance cameras, industrial controllers, switches, access points, cellular network base stations, and specialized edge routers, among others.

Fog computing does not replace the functions of cloud computing but complements them, and both coexist and cooperate with each other. These interactions are of different types since they can be between the same or different layers of the architecture or between different operators [11], which also brings challenges towards the establishment of a federation between both paradigms [12,14,17,18].

Integration between fog and cloud resources presents some challenges [15,18]:

- Scalability: The constant growth of geographically distributed devices represents a large amount of data that needs to be processed and analyzed. In this case, when IoT infrastructures exist, the distributed nature of fog computing is better suited than a centralized scheme such as cloud computing.
- Interoperability: This aspect has two major challenges. The first is related to a large number of protocols available in a heterogeneous IoT environment. The second is associated with the existence of several fog and cloud computing operators and their interaction. In this case, these computing paradigms are usually based on virtualization techniques deployed on platforms designed to run different applications in a homogeneous way, which can be an advantage when dealing with different applications, information sources, and operators.
- Real-time response: Bringing the computational resources that process data closer to the information sources is more crucial to carrying out activities that do not require in-depth analysis or high computing resources thanimproving the response time.
- Security: The amount of connected objects increases the difficulty of ensuring information security. In general terms, an integrated solution should aim to have general standard solutions that avoid the need to implement particular security mechanisms for each node.
- Environmental awareness: Many IoT applications rely on the capacity to know the context where the sensors are deployed. This can be enhanced by using distributed systems that are more aware of different environmental variables.
- Mobility: This is an essential requirement for the deployment of fog-computing-based schemes due to the growth of connected mobile devices. This represents a challenge in distributed systems since the solutions must maintain the required resources in a dynamic topology.
- Reliability: A solution of this nature must guarantee devices that operate correctly, keeping the communication and computing resources available and working.

Different surveys that address several aspects of fog computing and its integration with IoT and cloud computing have already been published. Surveys about the state of the art and research challenges can be found in [17,18,19,20,21], where several issues about fog computing are discussed, including architectural and security concerns and challenges. However, these articles do not focus on particular topics about fog computing. Instead, they include a review of several aspects related to these kinds of deployments, such as different types of architectures and integration with other paradigms. In [14], the authors considered the integration of fog-related technologies with smart cities; here, different use case scenarios are reviewed. Other initiatives collected alternatives for fog–cloud integration [22]. Further surveys [20] analyzed the implementation of new paradigms such as software-defined networking and network functions virtualization on IoT, and fog computing scenarios [23,24,25].

The resource allocation problem is a common challenge addressed in different fog computing surveys. However, not many of them analyze this issue in depth. In [15], the application placement problem was briefly described, and several options were classified according to the elected optimization metrics and the algorithms used to solve the problems. In this survey, there is no particular interest in analyzing the characteristics of each solution or the complexity of the considered architecture. In [26], the authors also provided an overview of the application placement problem in a fog environment and proposed a classification based on different scenarios and optimization strategies.

The contribution of this paper is twofold: first, we give a general definition of the application placement problem, focusing on the resource allocation aspect. We pay special attention to the resolution strategies and technical formulation of the problem, incorporating recent works that are not added in other surveys. Second, two recent research directions not covered in the reviewed works are included: the possibility of allocating resources in mobile computational nodes (e.g., vehicles) and the consideration of a federation between different fog operators and cloud operators.

This survey is structured as follows: Section 2 explains the methodology used to elaborate this review. Section 3 introduces the resource allocation problem in fog and cloud environments. Section 4 presents a taxonomy of different resource allocation alternatives according to control strategies, optimization objectives, resolution strategies, mobility support, and the inclusion of a federation between fog and cloud. Section 5 includes a discussion about the found research directions and challenges.

## 2. Methodology

This review concentrates on surveying different allocation strategies for applications deployed in nodes installed between the IoT and cloud layers, paying specific attention to strategies that assign resources in an online manner over dynamic topologies. Following the PRISMA guidelines, the methodology for building this report consists of three main phases:

### 2.1. Identification

We searched among the following databases: IEEE Xplore, ACM Digital Library, ScienceDirect, MDPI, SpringerLink, Google Scholar, and other engineering journals. We used the following keyword combination: (resource allocation or resource management) AND (fog computing or edge computing). This action delivered 1135 articles. In this phase, we removed 303 duplicated articles. The term “fog computing” was first coined by Cisco in 2012 [27], and the search conducted in the elected databases did not provide results focused on fog computing published earlier than the year 2016; articles before this year were oriented to cloud computing environments, a concept that is closely related to our research, but out of the scope of this survey. In this sense, articles published before 2016 were removed. We also removed articles that were written in languages different from English.

### 2.2. Screening

In this phase, three different researchers read the abstract and conclusions of each record. We excluded articles without experimental results and studies that did not evaluate at least one strategy for resource allocation in fog environments. Finally, we only included extended versions of the articles, removing, for example, conference papers that were further explored in their journal versions. After these actions, we retrieved 356 articles, obtaining 313 full texts to be read. At least two of the three reviewers read each article. Here, the researchers considered two reasons for final exclusions: the first one related to the characteristics of the strategy and the experimental scenario, and the second one associated with the experimental procedures conducted in the research. We considered distributed or centralized strategies that execute online resource allocation over dynamic topologies in works with detailed descriptions of their experimental environment. This is further explained in the following sections. Dissents were resolved by agreement.

### 2.3. Inclusion

**Figure 2:**
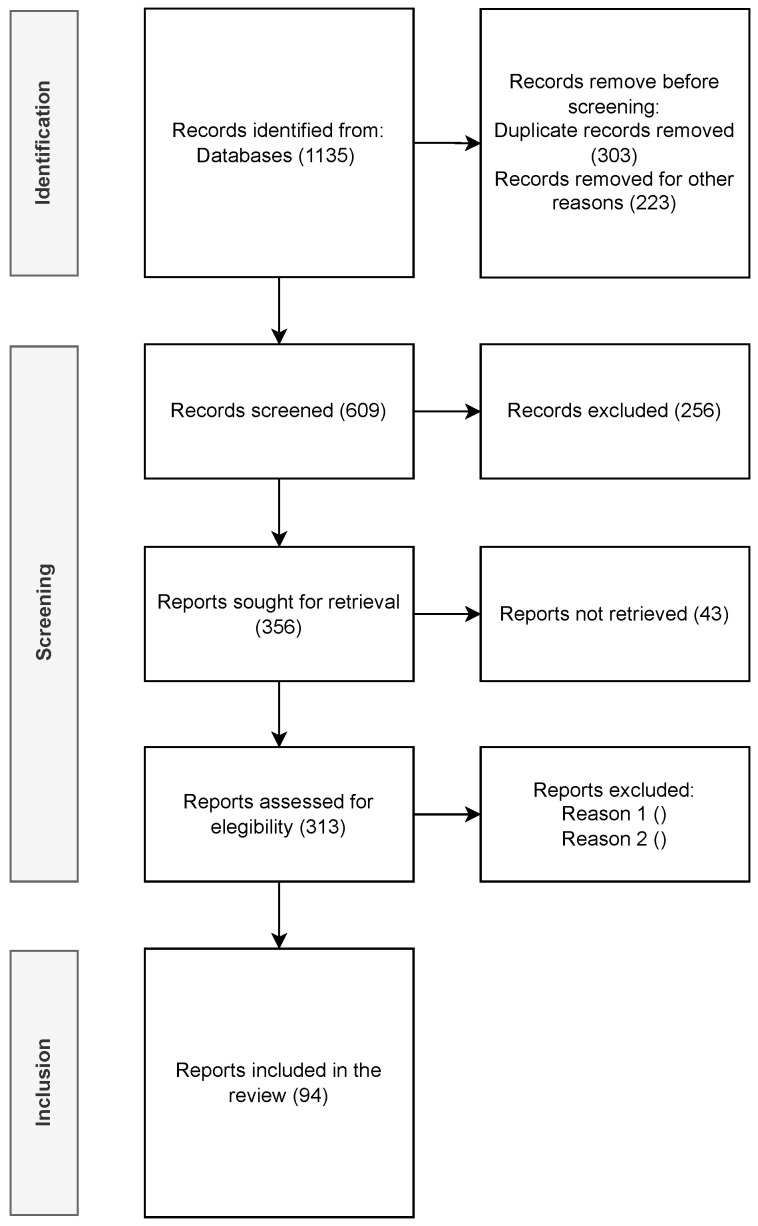
Methodology.

A total of 94 articles were classified according to the defined criteria and included in the survey. The methodology is summarized in Figure 2.

## 3. Resource Allocation in Fog/Cloud Environments

There are different problems related to the resource allocation problem in fog environments that are considered in the literature. They are usually known as the service or application placement problem [10,15,26]. This problem is quite similar to the problem related to virtual network functions embedding and can be described following the representation in [28].

**Figure 3:**
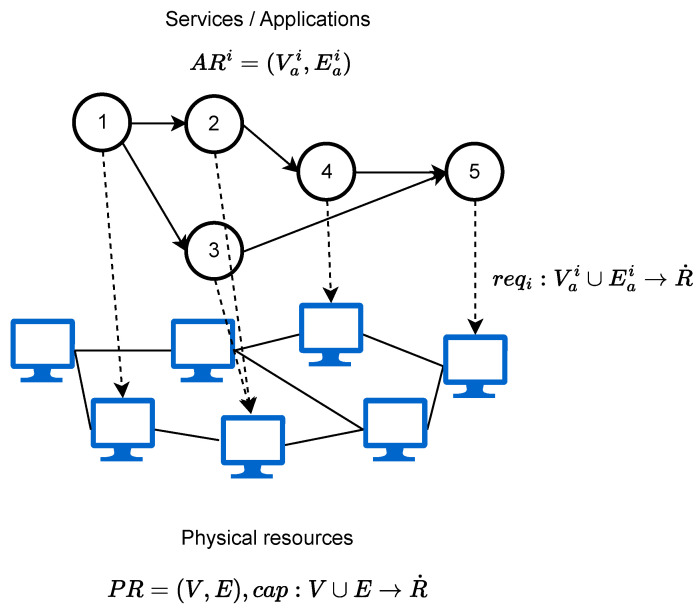
Resource allocationfor an application.

The terminology used in the following section is better described in Table 1. Let PR=(V,E) be a set of available physical resources where *V* represents the computational nodes and *E* the links between those nodes, and let $AR^{i}=\left(V_{a}^{i},E_{a}^{i}\right)$ be an application in a set of *n* application requests, where $V_{a}^{i}$ represents the set of functions of the application *i*, consuming computational capacity, and $E_{a}^{i}$ the flow of information between those functions, that consumes bandwidth. Let $\dot{R}=\prod_{j=1}^{m}R_{j}$ be a set of resource vectors, and let $cap:V\cup E\to \dot{R}$ be a function that assigns available resources to elements of the physical nodes. Ultimately, let $req_{i}:V_{a}^{i}\cup E_{a}^{i}\to \dot{R}$ be a function that assigns requests to elements of all application requests. Then, an application placement consists of two functions $f_{i}:V_{a}^{i}\to V$ and $g_{i}:E_{a}^{i}\to PR^{\prime }\subseteq PR$ for each $AR^{i}$ such that $\forall v^{i}\in V_{a}^{i}:req_{i}\left(v^{i}\right)\leq cap\left(f_{i}\left(v^{i}\right)\right)$ and $\forall e^{i}\in E_{a}^{i}:\forall e\in g_{i}\left(e^{i}\right):req_{i}\left(e^{i}\right)\leq cap\left(e\right)$. $PR^{\prime }$ is a subset of PR that represents a path inside the graph. Finally, we can define the residual physical resources, $RR=(V^{r},E^{r})$, that contain the available resources that are left after placing the set of applications $AR^{i}$ in PR. RR satisfies the following conditions: $\forall v\notin \left(f_{i}:V_{a}^{i}\to V\right):cap\left(v\right)=cap\left(v^{r}\right),v^{r}\in RR$ and $\forall e\notin g_{i}:E_{a}^{i}\to PR^{\prime }\subseteq PR:cap\left(e\right)=cap\left(e^{r}\right),e^{r}\in RR$. This means that the residual resources represent the remaining resources after application requests are placed. If RR has enough resources, then another application can be placed, repeating this process until no physical resources are left. Taking this into account is desirable to maximize the number of applications that are placed. In a more explicit way, the resource allocation problem consists in finding a representation of AR in PR in the presence of the defined constraints, as shown in Figure 3.

Virtual network functions embedding includes the joint placement of virtual services and traffic flows and has been thoroughly examined. Placing applications in the fog differs from VNF embedding since the latter assumes the possibility of programming network flows from a centralized controller. A fog application deployment can span through different providers that may not support centralized network management [15,29].

Some strategies concentrate on the distribution of resources in a single layer of the architecture [30,31]; however, in this work, we address strategies that consider resources in fog and cloud layers, including nodes with different characteristics, from those with low computing capacity up to micro data centers. The resource allocation strategies for the location of an application in an infrastructure can be classified according to the criteria used in [26]:

- Centralized (C) or distributed control (Di).
- Online (On) or offline resource assignment (Off).
- Static (S) or dynamic topology (Dy).
- Mobility supported (M) or mobility not supported (nM) (of fog or end nodes).

**Figure 4:**
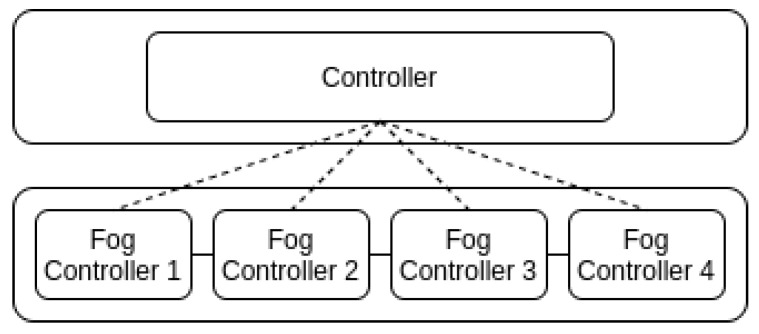
Distributed control.

**Figure 5:**
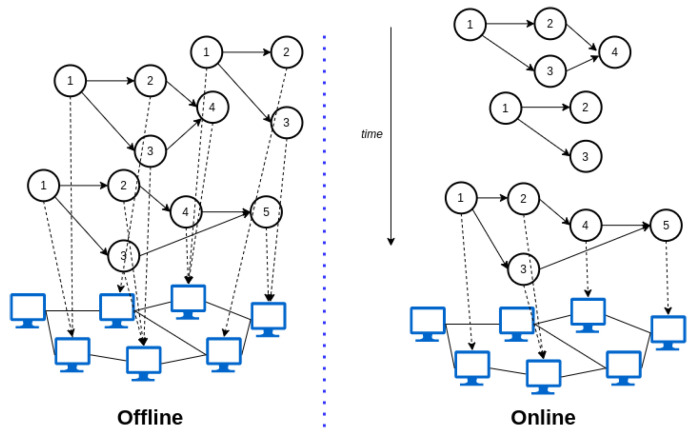
Offline or online provisioning.

**Figure 6:**
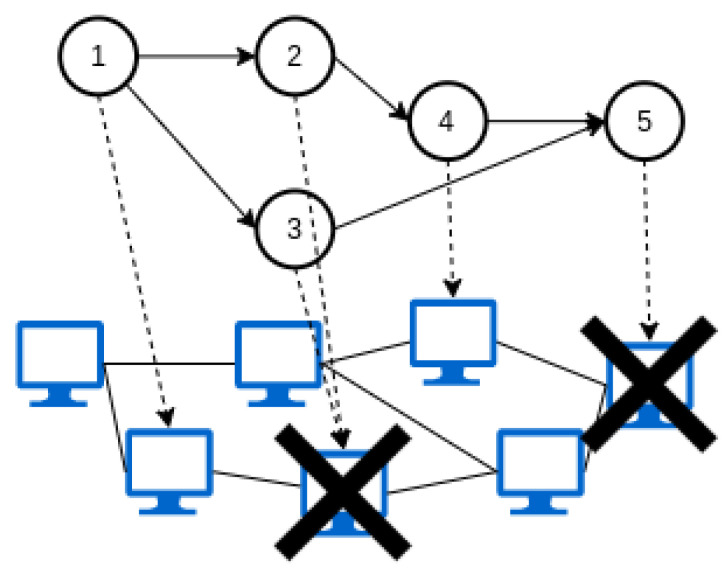
Static or dynamic topologies.

Strategies that are centralized rely on one entity that holds information about the whole topology. In contrast, distributed strategies consider that the decisions could be made by different controllers that manage groups of physical resources (see Figure 4). Another criterion is to consider that the decisions can be made with prior knowledge of the applications that must be deployed (offline) or the services will be provisioned as soon as they are demanded (online) as shown in Figure 5; the second alternative is far more difficult to implement and will sometimes lead to suboptimal solutions. In addition, the introduction of mobility of fog nodes (e.g., vehicles that act as fog nodes) or end users (e.g., information collected on smartphones) suppose that some physical resources would not always be available, creating a dynamic topology, forcing the controller to reassign the application to a new resource in order to maintain the service (see Figure 6, a representation of a dynamic topology).

In this survey, we concentrate on solutions of type **[C|Di]/On/Dy/M** and pay particular attention to multiobjective optimization strategies since they constitute a closer representation of a real deployment that includes an IoT/fog/cloud environment, where you can find online requirements of several applications that can be allocated in mobile fog nodes, showing a dynamic topology that can be affected when a node ends up without network coverage.

## 4. Taxonomy of Resource Allocation Strategies in Fog/Cloud Environments

This survey classifies the resource allocation strategies according to different criteria. As was stated before, we concentrate on strategies that consider online resource assignments on dynamic topologies. From this, we address different alternatives and analyze how each one of them assigns resources when placing applications.

### 4.1. Control Strategies

One of the first issues to address is the mapping coordination strategies. Two options are the most common along the different alternatives: centralized or distributed (see Figure 4). A centralized mapping requires the concentration of the global information in one point of the architecture and also the communications infrastructure to disseminate the decisions made by this entity. It could lead to finding a globally optimal solution. However, there could be issues related to scalability and computational complexity due to the amount of information that is exchanged. Most of the reviewed alternatives rely on centralized strategies. For example, the authors in [32] propose a unique entity that manages the fog and cloud resources in order to find the optimal distribution of the VM that will execute a part of a distributed application. This centralized entity implements a genetic algorithm in order to solve a multiobjective optimization problem, minimizing cost and latency and maximizing the number of applications deployed.

On the other side, a distributed control strategy allows multiple entities to control service mapping. These entities make decisions based on local information and use computational resources in their vicinity. These kinds of solutions are more flexible and probably more suitable to fog computing environments since they could be resilient to local changes and also can increase the system’s scalability. Still, they would probably lack information on the global system. These kinds of alternatives are often used in MEC environments [33,34,35], since particular devices make decisions related to computational tasks offloading in the architecture. As shown in Table 2, most of the reviewed papers implement centralized solutions. Still, in some works [36,37], the fog layer deals with tasks related to real-time processing and intermediate control entities that redirect the applications to the cloud (as shown in Figure 4). They propose a heuristic that takes local decisions and migrates the less requested services to cloud devices in the shortest path. An important thing to take into account when deploying a distributed control strategy is the communication protocol used for the information exchange between the fog controllers and, for example, a main controller in the cloud.

### 4.2. Optimization Objectives

The service placement problem is normally addressed from different optimization objectives, using different types of formulations and a variety of solving algorithms. Most of the papers in this survey try to optimize only one metric, while few alternatives propose simultaneously optimizing a group of metrics. Normally, when more than one metric must be optimized, the most adopted option is to include the other objectives as constraints. The most common optimization metrics are cost and latency, both desirable to minimize in most fog contexts used for real-time applications, where particular fog or cloud operators will compete to host IoT applications. In addition, it is common to find that latency is included as a constraint [60] since critical applications expose a time limit for completing a task.

Other habitual objectives are to minimize the computational resource utilization [60] while deploying the maximum number of services over the fog nodes; also reducing the monetary cost (for end users or for service providers), [36,51] associated with the data transmission or the computational consumption of the fog nodes and also to the deployment of a node; and even minimizing the energy consumption [40,80], which is one of the main concerns in IoT systems. The optimization objectives are summarized in Table 3. The resource allocation problem is normally formalized using integer programming (and its variants: integer linear programming, integer nonlinear programming, mixed-integer programming, and mixed-integer quadratic programming) or general constraint programming. Recently, novel approaches have considered formulations related to game theory, Markov decision processes, and reinforcement learning, an issue that is analyzed in the following section.

### 4.3. Resolution Strategies

Optimal resource allocation in fog environments is an NP-hard problem [26]. There are many issues that entangle this task; first, the heterogeneous nature and limited capacities of fog nodes, and also the dynamicity of the environment since the resources could appear or disappear instantly, and the sparse distribution of the fog nodes makes the resource allocation problem in fog networks a challenging task. In order to solve this problem, different types of solutions have been implemented, and five main approaches to solving the optimization problem are identified [26]:

- Exact solutions.
- Approximations.
- Heuristics.
- Metaheuristics (bio-inspired).
- Other alternatives: machine learning (DRL), game theory.

Exact solutions are the least common and focus primarily on small instances of the problem [36,68].

Because of the size and the various aspects related to fog infrastructures, heuristics and metaheuristics are often used; in works such as [19], a metaheuristic-based on Tabu Search was used in order to avoid suboptimal placements when minimizing the makespan and the communication cost. Several solutions implement genetic algorithms in order to define the best solution regarding a particular metric. For example, in [77], the authors compared the performance of a greedy first fit heuristic against a genetic algorithm where the chromosomes are vectors that represent a service placement plan containing the total number of services of all applications; they also contrasted scenarios where different combinations of resources (only fog, only cloud or fog, and cloud) are used, finding smaller delays when using the genetic algorithm.

Some recent papers include the implementation of machine learning and deep learning algorithms, such as in [45], where deep reinforcement learning (DRL) was used to adaptively allocate resources in order to reduce the average service time under an MEC environment that includes an SDN controller that runs the DRL agent. Here, the authors compared the performance of the DRL algorithm against a typical open shortest path first alternative, with different amounts of fog entities and applications to place. The DRL strategy obtained smaller service times and load distribution. A summary of the used resolution strategies can be found in Table 4.

### 4.4. Mobility Support

**Figure 7:**
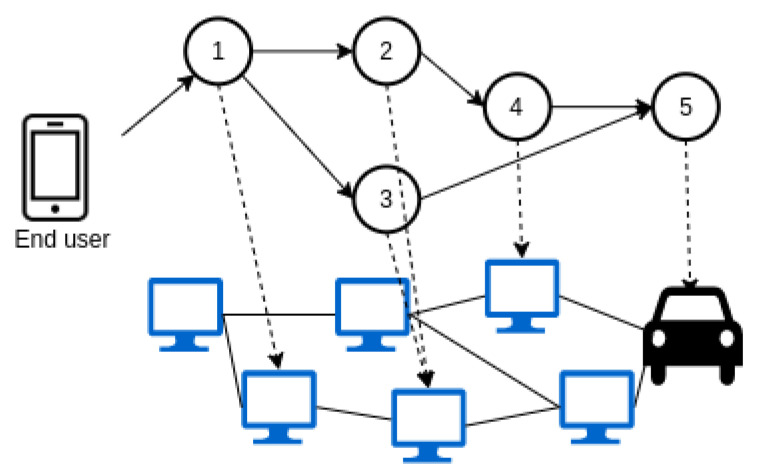
Mobility of fog nodes or end users.

Mobility management is a major issue in fog computing. Solutions that consider the mobility of end users are usually related to MEC environments. However, the mobility of fog nodes is not commonplace among the reviewed papers and is normally related to vehicular fog networks. Frequent changes in the position of fog nodes (see Figure 7) could lead to an excessive delay or packet loss or even service outages for an end user. In those situations, the orchestrator must be able to migrate the application to a new fog device. Some strategies consider the mobility of end users, but few authors tackle the issue of mobile fog nodes. In this case, considering mobile fog nodes implies that the physical topology where the applications are deployed is dynamic since, in some situations, some service (fog) nodes will be out of coverage. In [55], the authors introduce the concept of foglets, a programming infrastructure that provides an API for managing the application components deployed in the fog nodes. In this case, they consider geodistributed fog resources that can be managed by a centralized entity in charge of allocating resources according to a particular QoS requirement. The alternatives that explicitly state the use of mobile fog nodes in their implementations can be found in Table 5.

### 4.5. Federation between Fog and Cloud

The different needs held by heterogeneous applications could be fulfilled by a combination of resources in the fog and the cloud. For example, an application that needs more computational resources could be redirected to the cloud environment, whilst a routine that must be executed in a shorter time can experiment with better results if it is assigned entirely to fog nodes. Here, several configurations can be considered where, for instance, different operators on the cloud or the fog infrastructure, and even, in some cases, different service providers could manage their own fog infrastructure.

In every case, there is a challenge if the interaction between operators is needed. Federation among cloud operators is already a well-investigated issue [92,93], and usually presents problems related (and not limited) to the lack of formalization, to the deployment of architectures that can act in real-time and dynamic, online and distributed scheduling [94], topics that are already highlighted in this survey. Not many alternatives consider a federation between fog operators. However, in [32], the authors proposed an architecture that includes three different layers (cloud, federation, and application management) where a multiobjective optimization task was made, trying to minimize the cost, latency, and user’s footprint. Monetary cost is the most common metric to minimize in this kind of alternative since it is important to choose a provider that fulfills the application requirements at a minimal cost [38,48].

A summary of selected reviewed works is included in Table 6. They are classified according to their resolution and control strategies. All of them implement schemes of the type **[C|Di]/On/Dy/M**. The mobility parameter includes strategies that consider either mobile end users or mobile nodes. However, as seen before, few alternatives consider the latter. Most of them consider only one metric to be optimized, and initiatives that consider several optimization objectives usually choose one in the objective function and include the remaining metrics in the constraints of the problem. Many solution strategies are heuristics, and few authors have considered alternatives such as game theory or machine learning techniques. In addition, few authors have tackled the problem where multiple cloud or fog operators can host the same application.

### 4.6. Resilience in Fog and Cloud Environments

Resilience can be defined as the ability of a system (network) to provide and maintain an acceptable level of service in the presence of failures and different challenges during operation. Two principal fields concentrate the research on resilient networks: the first one tries to deal with how to design systems that, in the presence of problems, can keep the provision of a service, and the second one is related to guaranteeing that a system will behave as expected, taking into account measurable properties of the network (trustworthiness) [97]. Normally, cloud-based environments offer properties such as elasticity, virtualization, scalability, and geodistributed services that are often troublesome when trying to implement standard resilience alternatives [98]. In [99], some key aspects on which the resilience of fog computing systems depends were remarked upon. In our case, we concentrate on those points that relate to the resource allocation problem:

- Complexity: When network complexity increases, there is a higher probability of experimenting with unexpected failures. In fog computing systems, complex environments are common due to the number of devices and the heterogeneity of the hardware and functionalities. In addition, the chosen topology of a particular solution can lead to situations that are prone to random errors or security vulnerabilities. Therefore, the alternatives used for assigning resources in this kind of environment must consider how the architecture’s complexity can affect the availability of physical resources. In [100], the authors considered the existence of dependencies in complex systems and tried to characterize these relationships in order to reduce the level of intricacy of the network.
- Redundant resources: When assigning resources in fog computing systems, installing more nodes and reserving additional capacity to maintain the service in case of failure (without violating delay constraints) could increase the resilience of the network. In [101], the authors worked on a scenario of a vehicular network trying to minimize energy consumption. For this, they separated the fog nodes into clusters and enhanced energy saving by considering some collaboration between vehicular nodes.
- Deploying of agents: Distributed systems that implement different controllers can carry out actions to enforce problem mitigation. It also can improve system scalability in situations of high demand. In [102], a large-scale IoT network was managed by a distributed controller enhancing the recovery mechanism from a failure, supported by a distributed decision algorithm that rerouted the traffic to available nodes.

## 5. Research Directions and Challenges

This survey paper explored the existing literature on resource allocation in fog computing, including its various techniques and challenges. In this discussion section, we summarize our findings and offer insights into the current state of research and future directions in this field.

The proposed taxonomy classified resource allocation strategy according to different categories. We found that fewer solutions explore strategies based on distributed control and rely primarily on assigning this task to a central node. In addition, there is a growing interest in exploring scenarios of distributed applications over mobile fog nodes (similar to problems explored in vehicular networks). In terms of resolution strategies, tools such as ML and DL are paving the way toward becoming reliable alternatives for solving multiobjective optimization problems or working in more complex topologies; however, data gathering is still an issue to be attended to. Finally, there is still room for exploring resource allocation problems in environments with different operators. We will try to elaborate in a more detailed way on these aforementioned points.

Exploration of different control strategies: There is a lack of solutions that address the resource allocation problems in cloud/fog environments in a distributed way, relying on a single central node. This is mainly related to the fact that distributed algorithms are difficult to construct and implement due to the complexity of communication and synchronization. A distributed resource management strategy would need the implementation of a communication protocol between the control nodes [103]. Distributed control could be a well-fitted option to overcome issues related to scalability and reliability. For example, in SDN there is still a lot of research on designing efficient distributed control platforms since the structure of the control plane and the number and placement of multiple controllers critically impact the performance of a system [104].

Mobility of fog nodes: Due to the high mobility of end users and fog devices, the solutions must ensure the continuity of the service and consistent performance of the infrastructure, for example, by migrating in an appropriate way the allocated application to another chosen resource. This situation could become problematic when the researchers consider distributed applications instead of monolithic ones since the migration must consider communication between the parts of the application and latency between the components. In these cases, an approach with a predictive model for the users’ mobility could present advantages. Several studies propose models to predict the mobility of wireless sensor nodes [105]. In [106], a hidden Markov model is used to forecast the movement of the IoT devices in order to improve the handoff process and energy consumption.

Federation and multiple operators: Few alternatives consider the federation between fog and cloud or the possibility of having different fog operators, a situation that could increase the operational cost of the solution. This could be a common scenario since different actors could provide fog services; in this case, a solution that considers the monetary costs combined within a tolerable latency for a particular service is necessary. As stated before, there are several studies where a mesh of different cloud operators is considered. For example, one cloud operator that needs additional resources sends a request to foreign operators to enlarge its infrastructure elastically [107]. This is also an issue considered in the VNF placement problem, similar to in [108], where the authors implemented a multidomain orchestrator that manages resources in multiple operators. In [109], a deep reinforcement learning alternative was implemented in order to find an adequate VNF embedding in a noncooperative domain, where the network operators hid their infrastructure from other competing counterparts. In this sense, researchers could explore strategies that have proven their effectiveness in similar distributed problems such as VNF resource allocation.

Resilience in fog environments: Most of the reviewed alternatives do not consider failure protection in the proposed strategies. Most of them are related to architectures where vehicular networks are part of the edge and fog layers [66]. This is a crucial issue to be addressed due to the characteristics of fog nodes, which are typically energy- and availability-constrained. Resilient and survivable networks are points that have been exhaustively explored in NFV resource allocation problems and, in conjunction with SDN, are important alternatives to implement solutions that work in favor of the resilience of a fog computing system.

Finally, we found issues related to the experimentation and the formulation of the resource allocation problem, for which improvement could lead to a better understanding and tackling of the problem. Most of the reviewed papers are oriented to address the problem in a specific context and to be evaluated on different platforms, ending in the formulation of solutions that could not be compared. The configuration of nodes (number of fog nodes, cloud nodes, IoT devices) and characteristics of the deployment seem somewhat arbitrary. They could range from a few devices up to thousands of them. In addition, a variety of tools are used in a simulation of fog environments, where iFogSim [110] is probably the most common. Other simulators developed in common computational tools, such as Python or Matlab are also used. There is still room for working on a baseline in order to facilitate comparisons of different alternatives. Few alternatives implemented their solutions on real testbeds in order to analyze their execution and behavior. There are some efforts in implementing, for example, resource allocation strategies on commercial software such as Apache Spark or Hadoop [10], but in controlled environments with specific solutions. We also found that there is a lack of generality in formulating the resource allocation problem. Some papers, the majority found on MEC environments, consider monolithic applications, whilst the most elaborated alternatives represent the deployment of an application or a service as a directed acyclic graph, where the nodes are functions that take part in the processing of the information or can also be sources or destinations of the data. Edges represent the flow of information between nodes. The latter representation could be a better method of representing the problem.

## 6. Conclusions

This paper focuses on the resource allocation problem in the IoT and cloud layers continuum, an issue with open challenges and discussions. This paper collected several works on this topic, proposing a taxonomy to classify the different alternatives according to the control strategies, optimization objectives, resolution strategies, mobility support, and federation between fog and cloud paradigms. This article focuses on strategies that implement online resource allocation on dynamic topologies.

There are several approaches to resource allocation, including centralized or distributed control and online or offline resource assignation, and even strategies that seek to improve resilience on the network. In addition, different environmental assumptions could be considered, such as dynamic topologies that include mobile nodes or scenarios that pose integration between fog and cloud operators. These resource allocation problems are usually solved using exact methods in smaller scenarios or heuristics and metaheuristics on larger layouts. Recent works have focused on proving the effectiveness of game-theoretic models or deep reinforcement learning alternatives.

Despite significant progress in resource allocation research, many challenges and open research questions still need to be addressed. For example, one crucial issue we seek to address in the future is evaluating different algorithms in the scenarios considered in this research.

This paper also formally defines the resource allocation problem in these environments. In conjunction with the assembled taxonomy, we aim to ease access to works developed in this specific context and to identify the challenges in this topic.

## Figures and Tables

**Table 1:** Terminology.

Term	Description
PR=(V,E)	PR is a set of V nodes and E links that hold the physical resources, representing the computational resources and connectivity between nodes.
$AR^{i}=\left(V_{a}^{i},E_{a}^{i}\right)$	AR is the $i^{th}$ application request consisting of $V_{a}^{i}$ functions that take part in the information processing of the application (that consume computational capacity, e.g., RAM memory, CPU, or storage), and $E_{a}^{i}$ represents the flow of the information (in bits per second, for example).
$RR=(V^{r},E^{r})$	Represents the nodes that hold residual resources after placing the application requests in the available physical resources.
$\dot{R}=\prod_{j=1}^{m}R_{j}$	$\dot{R}$ contains the resource vectors for all resources (physical and logical).
$cap:V\cup E\to \dot{R}$	The function cap assigns a capacity to an element of the physical resources (node or link).
$req_{i}:V_{a}^{i}\cup E_{a}^{i}\to \dot{R}$	The function $req_{i}$ assigns an application request to an element of $AR^{i}$ (node or link).
$f_{i}:V_{a}^{i}\to V$	$f_{i}$ is a function that maps a part of the $i^{th}$ application to a physical node.
$g_{i}:E_{a}^{i}\to PR^{\prime }\subseteq PR$	$g_{i}$ is a function that maps a link of the application request to a path of PR.

**Table 2:** Classification according to control strategies.

Control Strategies	References
Centralized control	[15,19,30,32,35,38,39,40,41,42,43,44,45,46,47,48,49,50,51,52,53,54,55,56,57,58,59,60,61,62,63,64,65,66,67,68,69,70,71,72,73,74,75,76]
Distributed control	[33,34,36,37,53,77,78,79,80,81,82,83,84,85,86,87,88]

**Table 3:** Classification according to optimization objectives.

Optimization Objective	References
Cost	[15,19,30,32,33,34,35,36,38,41,43,44,49,53,56,57,60,63,67,71,73,75,76,78]
Latency	[19,32,34,45,48,51,52,53,55,59,62,64,70,74,79,81,84,88]
Link utilization—throughput	[30,38,42,43,60,85,87,89,90,91]
Hops—distance	[37,51,54,61]
Energy consumption	[39,40,42,46,47,50,66,68,72,80,84,86]
Usage of fog resources—application placements	[33,60,61,65,77,81,85,88]
Other	[15,60,66,69,72,82]

**Table 4:** Classification according to resolution strategies.

Resolution Strategy	References
Heuristic	[15,36,37,39,41,42,43,44,47,48,49,50,51,55,56,59,63,64,65,67,69,70,71,72,74,77,79,80,82,83]
Metaheuristic	[19,66,88]
Game theory	[30,34,38,73,75,76,78,86]
Genetic algorithms (metaheuristic)	[32,40,46,60,61,62,66,77]
RL-DRL	[33,45,57,74,81,84,85,87,91]
MDP—Lyapunov optimization	[52,53,58]

**Table 5:** Mobility support of reviewed alternatives.

Mobility Support	Related Work
Fog nodes	[19,34,44,66,73,74,84,87,88]
End nodes	[19,33,35,36,45,47,52,53,55,57,58,68,69,70,71,72,75,76,81,85,86]

**Table 6:** Reviewed works on resource allocation.

Resource Assignation in Fog–Cloud Environments						
Related Work	Objective	Control	Mob.	Strategy	Fed	Contribution
Dynamic application deployment in federations of clouds and edge resources using a multiobjective optimization AI algorithm [32]	Cost, latency, users	**C**		Genetic algorithm	✓	Presented a resource allocation on a federated architecture based on a genetic algorithm.
A Dynamic Service-Migration Mechanism in Edge Cognitive Computing [57]	Cost	**C**	✓	Reinforcement learning		Proposed a dynamic service migration and deployed over a physical scenario and solved the service placement using RL.
A proximal algorithm for joint resource allocation and minimizing carbon footprint in geodistributed fog computing [80]	Energy consumption	**D**		Heuristic		Considered the problem of joint resource allocation and minimizing carbon footprint problem for video streaming service in fog computing. Developed a distributed algorithm based on the proximal algorithm and alternating direction method of multipliers (ADMM).
Towards dynamic resource provisioning for IoT application services in smart cities [95]	Users, bandwidth, hops	**C**		Exact methods		Developed a resource discovery service to exchange resource allocation information between the fog and the cloud layer.
Efficient Placement of Multicomponent Applications in Edge Computing Systems [56]	Cost	**C**		Heuristic		Addressed the problem of placement of multicomponent applications in MEC, and developed a heuristic algorithm based on an interactive matching process.
MigCEP: Operator Migration for Mobility-Driven Distributed Complex Event Processing [36]	Cost	**Di**	✓	Heuristic	✓	Presented a method to reduce cost in a federated environment (multiple fog operators), where the migrations are planned ahead of time.
Incremental Deployment and Migration of Geo-Distributed Situation Awareness Applications in the Fog [55]	Latency	**C**	✓	Heuristic		They propose foglets, which are APIs for storing and retrieving data on the local nodes and enabling communication among the resources in the fog and cloud.
Service placement for latency reduction in the Internet of Things [54]	Hops	**C**		Heuristic		Proposed a service placement architecture for the Internet of Things.
A Fog-based Architecture and Programming Model for IoT Applications in the Smart Grid [79]	Latency	**C**		Heuristic		Introduced fog computing coordinator, which manages computing nodes of IoT applications in the smart grid.
Follow Me at the Edge: Mobility-Aware Dynamic Service Placement for Mobile Edge Computing [53]	Cost, latency	**C, Di**	✓	MDP—Lyapunov opt.		Applied Lyapunov optimization to decompose the long-term optimization problem into a series of real-time optimization problems to handle user mobility.
Dynamic service migration and workload scheduling in edge–clouds [52]	Latency	**C**	✓	MDP - Lyapunov opt.		Applied a new approach to solving constrained MDP to a dynamic service migration and workload scheduling in edge–clouds environments.
Elastic urban video surveillance system using edge computing [50]	Distance, latency	**C**		Heuristic		Designed a three-tier edge computing system NFV-SDN architecture to elastically adjust computing capacity and dynamically route data to proper edge servers for the real-time surveillance applications.
Optimal Workload Allocation in Fog–Cloud Computing Toward Balanced Delay and Power Consumption [51]	Energy consumption	**C**		Heuristic		Tackled a workload allocation problem by decomposing the primal problem into three subproblems.
Joint Optimization for Task Offloading in Edge Computing: An Evolutionary Game Approach [78]	Latency, cost	**C**		Game theory		Proposed a resource-allocation strategy based on evolutionary game theory to deal with task offloading to multiple heterogeneous edge nodes and central clouds among multi-users.
Optimized Provisioning of Edge Computing Resources With Heterogeneous Workload in IoT Networks [49]	Cost, latency	**C**		Heuristic		Formulated the problem of resource provisioning and workload assignment for IoT services to jointly decide on the number and the location of edge servers and applications to deploy, using a decomposition approach to divide the original problem into two subproblems.
Optimal Placement of Cloudlets for Access Delay Minimization in SDN-Based Internet of Things Networks [48]	Latency	**C**		Heuristic		Investigated the optimal placement of cloudlets using SDN to minimize the average access delay.
UCAA: User-Centric User Association and Resource Allocation in Fog Computing Networks [47]	Latency, energy consumption	**C**	✓	Heuristic		Presented a user-centric resource allocation scheme, trying to minimize a utility function that depends on several parameters.
Optimizing QoS-Assurance, Resource Usage and Cost of Fog Application Deployments [15]	Latency, cost, QoS	**C**		Heuristic		Developed a prototype that runs a multiobjective optimization framework to determine the deployments of the application that provide the best tradeoff among optimization objectives.
Task-Driven Resource Assignment in Mobile Edge Computing Exploiting Evolutionary Computation [46]	Latency, energy consumption	**C**		Genetic algorithms		Proposed a joint optimization problem for task-driven resource assignment based on evolutionary computation over three typical task-driven cases.
A lightweight decentralized service placement policy for performance optimization in fog computing [37]	Hops	**Di**		Heuristic		Proposed a decentralized optimization policy for service placement in fog computing addressed to place the most popular services as close to the users as possible.
Smart Resource Allocation for Mobile Edge Computing: A Deep Reinforcement Learning Approach [45]	Service time	**C**		Reinforcement learning		Proposed a deep reinforcement learning-based resource allocation scheme, to allocate computing and network resources adaptively.
Fog Vehicular Computing: Augmentation of Fog Computing Using Vehicular Cloud Computing [89]	Latency	**C**	✓	-		Proposed fog vehicular computing (FVC) to augment the computation and storage power of fog computing. In addition, designed a comprehensive architecture for FVC.
A cost- and performance-effective approach for task scheduling based on collaboration between cloud and fog computing [43]	Cost	**C**		Heuristic	✓	Proposed a scheduling algorithm to achieve the balance between the performance of application execution and the mandatory cost for the use of cloud resources.
Energy and time efficient task offloading and resource allocation on the generic IoT–fog–cloud architecture [42]	Energy consumption	**C**		Heuristic	✓	Proposed a general IoT–fog–cloud architecture, and resource allocation was formulated into the energy and time cost minimization problem.
Application Component Placement in NFV-Based Hybrid Cloud/Fog Systems With Mobile Fog Nodes [19]	Latency, cost	**C**	✓	Metaheuristic	✓	Used the random waypoint mobility model for fog nodes and proposed a Tabu-Search-based component placement (TSCP) algorithm to find suboptimal resource placements.
A hybrid approach to scheduling real-time IoT workflows in fog and cloud environments [41]	Cost	**C**		Heuristic	✓	Proposed a hybrid fog- and cloud-aware heuristic for the dynamic scheduling of multiple real-time Internet of Things (IoT) workflows in a three-tiered architecture.
Workload Allocation in IoT–Fog–Cloud Architecture Using a Multiobjective Genetic Algorithm [90]	Latency, energy consumption	**C**		Genetic algorithms	✓	Formulated an alternative to maintain a trade-off between energy consumption and delay in processing workloads in fog.
Energy-efficient task allocation and energy scheduling in green energy powered edge computing [90]	Energy consumption	**C**		Heuristic	✓	Investigated the energy cost minimization problem with joint consideration of VM migration, task allocation, and green energy scheduling and proved its NP-hardness.
Migration Modeling and Learning Algorithms for Containers in Fog Computing [91]	Energy consumption, latency	**C**	✓	Reinforcement learning		Proposed container migration algorithms and architecture to support mobility tasks with various application requirements. Modeled such container migration strategy as multiple dimensional Markov decision process (MDP) spaces.
Computing Resource Allocation in Three-Tier IoT Fog Networks: A Joint Optimization Approach Combining Stackelberg Game and Matching [38]	Cost	**C**		Game theory	✓	Proposed a joint optimization framework for fog nodes, service providers, and users to achieve the optimal resource allocation schemes in a distributed fashion.
A Hierarchical Game Framework for Resource Management in Fog Computing [30]	Cost, latency	**C**		Game theory	✓	Proposed a three-layer hierarchical game framework to solve the problem related to the resource allocation in the virtualized network, the asymmetric information problem, and the resource matching in the physical network.
Trust-Oriented IoT Service Placement for Smart Cities in Edge Computing [60]	Resource utilization, load balancing, variance, cost	**C**		Genetic algorithm		Proposed a modification of Pareto evolutionary algorithm to improve the edge node performance.
Dynamic On-Demand Fog Formation Offering On-the-Fly IoT Service Deployment [61]	Service deployed, QoS, Availability, hops, distance	**C**		Genetic algorithm		Proposed an evolutionary memetic algorithm to solve a multiobjective container placement optimization problem.
Optimized Placement of Scalable IoT Services in Edge Computing [62]	Latency	**C**		Genetic algorithm		Jointly treated the load distribution and placement of scalable IoT services, to minimize the potential violation of their QoS requirements due to the limitations of edge computing resources.
Topology-Aware Resource Allocation for IoT Services in Clouds [83]	Link utilization	**C**		Heuristic		Investigated the VM placement problem for balanced network utilization by avoiding network congestion.
Placement and Chaining for Run-Time IoT Service Deployment in Edge-Cloud [96]	Cost	**Di**	✓	Heuristic	✓	Presented an NFV-based high-level architecture for a system that enables the deployment of IoT services across multiple edges and clouds.
Towards Network-Aware Resource Provisioning in Kubernetes for Fog Computing Applications [64]	Latency	**C**		Heuristic		Studied the VNF optimal placement problem in NFV-based edge cloud systems with IoT nodes. Considered IoT service chains composed of multiple VNFs deployed on edge clouds.
IoT Application Placement Algorithm Based on Multidimensional QoE Prioritization Model in Fog Computing Environment [82]	QoE	**Di**		Heuristic		Presented a 2-phase IoT application placement algorithm based on the multidimensional QoE (MD-QoE).
Optimization of Service Placement with Fairness [65]	Node usage, fairness	**C**		Heuristic		Presented an architecture composed by a fog and a cloud layer, where the fog contains a set of independent clusters. Proposed a heuristic to maximize fog usage and fairness.
Optimized IoT Service Placement in the Fog [77]	Number of app placements	**C**	✓	Genetic algorithm		Presented a conceptual fog computing framework, and modeled the resource allocation problem considering the heterogeneity of applications and resources.
When Deep Reinforcement Learning Meets Federated Learning: Intelligent Multitimescale Resource Management for Multiaccess Edge Computing in 5G Ultra Dense Network [81]	Latency, network usage	**Di**	✓	Reinforcement learning		Presented a 2-timescale DRL approach to jointly optimize execution time and network resource usage in an ultradense edge computing environment.
Virtual Service Placement for Edge Computing Under Finite Memory and Bandwidth [58]	Throughput	**C**	✓	Lyapunov optimization		Jointly optimized the service placement, data admission, and resource allocation of an edge server to maximize the time-average service throughput of the server.
Near Real-Time Optimization of fog Services placement for responsive Edge computing [59]	Latency	**C**		Heuristic		Presented a service scheduling algorithm for fog and edge networks containing hundreds of thousands of devices, which is capable of incorporating changes in network conditions and connected devices.
Resource Allocation in 5G IoV Architecture Based on SDN and Fog–Cloud Computing [59]	Delay, stability, energy consumption, load balancing	**C**	✓	Genetic algorithm	✓	Proposed a multiobjective optimization problem solved via a modified GA in order to improve resource allocation in a vehicular network combined with cloud resources.
A Micro-Level Compensation-Based Cost Model for Resource Allocation in a Fog Environment [59]	Cost	**C**		Heuristic		Proposed a heuristic for resource allocation trying to minimize the cost of placing applications and compared its performance against the best-fit algorithm, obtaining better results in terms of cost, successful placements and delay.
Blockchain-Based Edge Computing Resource Allocation in IoT: A Deep Reinforcement Learning Approach [33]	Cost, number of app placements	**Di**	✓	Reinforcement Learning, Markov decision process		Presented a general framework for blockchain-based edge computing scenarios. Used a reinforcement learning algorithm (AC3) to solve the resource (contract) assignation problem that is formulated as an SMDP.
Computation Offloading and Resource Allocation For Cloud Assisted Mobile Edge Computing in Vehicular Networks [34]	Delay, cost	**Di**	✓	Game theory		Presented a distributed strategy for computation offloading and resource allocation in vehicular networks, based on game theoretic approach.
Computation Offloading and Resource Allocation in Wireless Cellular Networks With Mobile Edge Computing [35]	Cost	**C**	✓	Exact methods		Presented an ADMM decentralized algorithm for computation offloading, resource allocation, and content caching, in order to maximize the revenue of an MEC operator.
Contract-Based Computing Resource Management via Deep Reinforcement Learning in Vehicular Fog Computing [84]	Latency, energy consumption	**Di**	✓	Deep reinforcement learning		Presented a resource management scheme based on contract theory and used a DRL method to implement the strategy.
Cooperative Computation Offloading and Resource Allocation for Blockchain-Enabled Mobile-Edge Computing: A Deep Reinforcement Learning Approach [85]	Computation rate, throughput	**D**	✓	MDP, Deep reinforcement learning		Proposed a blockchain MEC system, where the offloading and resource allocation problem is jointly solved.
Decentralized Computation Offloading and Resource Allocation for Mobile-Edge Computing: A Matching Game Approach [86]	Energy consumption	**D**		Game theory		Proposed a strategy to jointly determine computation offloading, transmit power, and resource allocation, in a HetNet scenario, using a matching game formulation.
Deep Reinforcement Learning for Offloading and Resource Allocation in Vehicle Edge Computing and Networks [87]	Network utilization	**Di**	✓	Deep reinforcement learning		Proposed a resource assignation and offloading strategy in an MEC environment with vehicles as intermediate edge servers.
Green Large-Scale Fog Computing Resource Allocation Using Joint Benders Decomposition, Dinkelbach Algorithm, ADMM, and Branch-and-Bound [68]	Energy consumption	**C**		Exact methods		Proposed a large-scale MINLP problem and solved it by dividing it into two subproblems. Tried to maximize a utility function based on energy consumption.
Fog Computing for 5G Tactile Industrial Internet of Things: QoE-Aware Resource Allocation Model [69]	Blocking probability	**C**		Heuristic		Proposed a QoS-aware model for resource allocation in a fog environment.
Joint Task Assignment, Transmission, and Computing Resource Allocation in Multilayer Mobile Edge Computing Systems [70]	Latency	**C**		Heuristic		Proposed a multilayered dataflow processing system, where the resource allocation problem was solved using a heuristic.
MOERA: Mobility-Agnostic Online Resource Allocation for Edge Computing [71]	Cost	**C**	✓	Heuristic		Proposed an online resource allocation model in a mobile edge environment, validating it with real and synthetic data.
Joint communication and computing resource allocation in vehicular edge computing [88]	Latency, completed tasks	**Di**	✓	Metaheuristic		Introduced MEC technology to VANET, providing resources from vehicles in the road.
PORA: Predictive Offloading and Resource Allocation in Dynamic Fog Computing Systems [72]	Energy consumption, queue length	**C**	✓	Heuristic		Proposed a predictive offloading scheme in fog computing environments for resource assignation.
Multiattribute-Based Double Auction Toward Resource Allocation in Vehicular Fog Computing [73]	Cost	**C**	✓	Game theory		Proposed an auction mechanism for resource allocation in a vehicle fog computing network.
Resource Allocation for Vehicular Fog Computing Using Reinforcement Learning Combined With Heuristic Information [74]	Latency	**C**	✓	Deep reinforcement learning, heuristic		Proposed a combined strategy (DRL + heuristic) to solve the resource allocation problem in a vehicular fog network.
Three Dynamic Pricing Schemes for Resource Allocation of Edge Computing for IoT Environment [75]	Cost	**C**	✓	Game theory		Proposed different dynamic pricing schemes in an MEC environment, in order to assign offloading computational resources.
Wireless and Computing Resource Allocation for Selfish Computation Offloading in Edge Computing [76]	Cost	**C**	✓	Game theory		Proposed a joint allocation of wireless and computing resources, with devices that decide by themselves whether to offload their computing tasks.

## Data Availability

No new data were created or analyzed in this study. Data sharing is not applicable to this article.
